# Depression among Ethiopian Adults: Cross-Sectional Study

**DOI:** 10.1155/2016/1468120

**Published:** 2016-05-09

**Authors:** Getasew Legas Molla, Haregwoin Mulat Sebhat, Zebiba Nasir Hussen, Amsalu Belete Mekonen, Wubalem Fekadu Mersha, Tesfa Mekonen Yimer

**Affiliations:** ^1^Debre Tabor Hospital, Debre Tabor, Ethiopia; ^2^Psychiatry Department, University of Gondar, P.O. Box 196, Gondar, Ethiopia; ^3^Amanuel Mental Specialized Hospital, P.O. Box 1971, Addis Ababa, Ethiopia; ^4^College of Health Sciences, Debre Tabor University, P.O. Box 272, Debre Tabor, Ethiopia; ^5^College of Medicine and Health Sciences, Wolaita Sodo University, P.O. Box 138, Wolaita Sodo, Ethiopia

## Abstract

*Background*. Depression is one of the most common mental disorders worldwide and is the second leading cause of disability and major contributor to suicide.* Methods*. Community based cross-sectional study was conducted among 779 adults residing in Northwest Ethiopia. Multistage cluster sampling technique was used to select study participants. Depression was measured by Patient Health Questionnaire (PHQ-9). Bivariate as well as multivariate logistic regressions were used to identify associated factors. *p* value of < 0.05 was considered statistically significant.* Result*. The prevalence of depression was 17.5%, where 10.7% of patients were with mild depression, 4.2% were with moderate depression, 1.9% were with moderately severe depression, and 0.6% had severe depression. Being female, age of 55 years and above, poor social support, having a comorbidity medical illness, current tobacco smoking, and living alone were significantly associated with depression.* Conclusion and Recommendation*. The prevalence of depression in Ethiopia is as common as the other lower and middle income countries. Female gender, being currently not married, poor social support, low wealth index, tobacco smoking, older age, having comorbid illness, and living alone were significantly associated with depression. So, depression is a significant public health problem that requires a great emphasis and some factors like smoking habit are modifiable.

## 1. Background

Depression is a common mental disorder characterized by depressed mood, loss of interest or pleasure, feeling of worthlessness or inappropriate guilt, disturbed sleep and appetite, feeling of tiredness, and poor concentration for a minimum of two weeks [1]. It affects 350 million people in the world and is the second leading cause of disability. The mortality rate due to suicide is 20 times greater among depressed individuals than the general population [2–4].

The magnitude of depression varies across different regions. WHO health survey conducted among 245,404 adults of 60 courtiers indicated that the overall one-year prevalence of depression was 3.2% [5]. Eighteen-country adult based study reported that the life time prevalence of Major Depressive Disorder (MDD) was 14.6% in 10 high income countries and 11.1% in 8 low and middle income countries [6]. Depression was also reported as 4.8% in Canada [7], 11.1% in Estonian [8], 15.1% in India, and 45.6% in Pakistan [9, 10] and it ranges from 4.9% to 29.3% in different African countries including Ethiopia [11–13].

According to CDC mortality and morbidity report in US, depression was more common in females (4.0%) than males (2.7%) and in unemployed persons (9.8%) than employed persons (2.0%) [14]. Depression was also common among substance users, disrupted marital status, older age, and lower educational level [15–19]. Other factors associated with depression in different studies were lack of social support, rural residence, having chronic illness, smoking, and alcohol use [20–23].

Assessing the prevalence of depression and the associated factors is very important in order to give good impression for the policy makers and to provide an input for evidence based intervention of depression. It is also helpful to indicate the modifiable factors that can affect depression for clinicians.

## 2. Methods

Community based cross- sectional study was conducted in 2015 at Ebinat town, Northwest Ethiopia. The town is located in south Gondar, Ethiopia, which is 680 km far from Addis Ababa, and the main livelihood is farming.

The sample was calculated using single population proportion formula by assuming the prevalence of depression of 9.1%, margin of error of 3%, 95% confidence level, design effect of 2, and 10% nonrespondent rate which gives a total sample of 779. Participants were recruited by using cluster sampling technique. All individuals whose age was greater or equal to 18 years were included in the study.

The dependent variable was depression and sociodemographic characteristics (age, sex, marital status, educational status, occupational status, wealth index, and residence), clinical factors (comorbid medical or surgical illness, past history of mental illness, and family history of mental illness), substance related factors, and social support were independent variables.

In this study participants were considered to have depression if their PHQ-9 score was greater or equal to 5 (5–9 mild depression, 10–14 moderate depression, 15–19 moderately severe depression, and 20–27 severe depression). Social support was described by sum score of Oslo 3 social support scale (OSS-3) as strong social support (12–14), intermediate social support (9–11), and poor social support (3–8).

The data were collected by pretested structured interviewer administered questionnaire which includes sociodemographic characteristics, substance use, clinical factors, social support (OSS-3), and PHQ-9. PHQ-9 screening tool has nine items and was validated in Ethiopia with sensitivity of 86% and specificity of 67% [24]. To maintain the quality of data training was given for data collectors and supervisors. The collected data were checked on daily basis for completeness and consistency. The data were entered in to Epi-Info version 7 and exported to SPSS-20 for analysis. Descriptive statistics was used to describe the study population in relation to relevant variables. Both bivariate and multivariate logistic regression models were used to identify associated factors. Odds ratio and their respective 95% confidence intervals were computed and variables with *p* value less than 0.05 were considered significantly associated.

Ethical clearance was approved by the Institutional Review Board (IRB) of University of Gondar and Amanuel Mental Specialized Hospital. After the purpose and related issues of the study were clearly informed, verbal consent was obtained from each study participant. Confidentiality was maintained by using anonymous questionnaire and by keeping the data in secure place. Participation was voluntary based and participants had the right to refuse the interview whenever they want. Participants who were found severely depressed were linked to the nearby health facility.

## 3. Results

A total of 779 residents of the town were included in the study. Among the respondents 416 (53.4%) were male. Majority of participants were in the age group of 25–34 years with median age of 28 years and interquartile range of 14. About 373 (47.9%) were married and 424 (54.1%) had educational status of secondary school and above (Table 1).

From the total study participants, 62.3% of them drink alcohol, 9.5% of them chew khat, and 1.9% of the participants smoke tobacco products at least once in the last three months. Regarding life time use of substances 78%, 17.2%, and 7.6% of the participants consume alcohol, khat, and tobacco, respectively.

Fifty-three (6.8%) participants had reported comorbid medical illness, where 20 (2.6%) had HIV/AIDS, 8 (1%) had hypertension, 6 (0.8%) had diabetic mellitus, 5 (0.6%) had renal disease, and the remaining ones had other disorders.

Regarding social support, 278 (35.7%) had poor social support, 345 (44.3%) of the participants had intermediate social support, and 156 (20%) of participants had strong social support.

Among participants, 136 had score of ≥5 in PHQ-9 which makes the prevalence of depression 17.5% (mild depression 10.7%, moderate depression 4.2%, moderately severe depression 1.9%, and severe depression 0.6%).

Age, wealth index, sex, marital status, occupation, educational status, social support, current tobacco smoking, having comorbid illness, and living circumstance were associated with depression in bivariate analysis.

However, older age, lower wealth index, being divorced and widowed, being female, poor social support, having comorbid illness, tobacco smoking, and living alone were significantly associated with depression in multivariate analysis (Table 2).

## 4. Discussion

The study showed that the prevalence of depression was 17.5%. The finding of this study was in line with the finding in urban India which was 15.1% and a study conducted in 18 countries (14.6%) [6, 9]. The prevalence was lower than studies done in Pakistan (45.98%) and Uganda (29.3%) [10, 12]. However, it was higher than the studies done in USA (9%), Canada (4.8%), Korea (11%), Malaysia (10.3%), Estonia (11.1%), and South Africa (9.7%) [7, 8, 11, 16, 18].

People in the age group of 55 years and above had 5.21 times more odds to develop depression than people less than 25 years. The possible reasons may be inability to perform daily activities, sedentary life style, and occurrence of concomitant medical illness. The finding was supported by studies done in Korea, USA, and Ethiopia [13, 14, 16]. The odds of having depression among participants with low income were 2.19 times more as compared with their counterparts. The finding was consistent with the Korean study [16]. Participants who were currently not married (divorced and widowed) were 2.6 times more likely to have depression as compared with participants who were married.

Females were 2.45 times more likely to be depressed as compared with males. This may be due to having fluctuating hormone levels that are associated with symptoms of depression. This result is supported by studies done in USA, Pakistan, Nigeria, South Africa, Uganda, and Ethiopia [6, 11–13, 16]. Depression was more common among participants who have poor social support as compared with individuals who have good social support. The finding was supported by a study done in Japan [17].

Comorbid medical illnesses were significantly associated with depression. The possible explanation may be that medical illness can cause tremendous life changes which may limit mobility and independency, interferes with doing enjoyable activities, and consequently decreases self-confidence that results in depressive symptoms. The result was supported by other studies done in Malaysia and Ethiopian [13, 18]. The odds of having depression for participants who smoke tobacco were 5.67 times more than those who did not smoke tobacco. This finding was supported by a study conducted in Australia [20].

## 5. Conclusion

The prevalence of depression in Ethiopia is as common as the other lower and middle income countries. The study also revealed that female gender, being currently not married, poor social support, low wealth index, tobacco smoking, older age, having comorbid illness, and living alone were significantly associated with depression. So depression is a significant public health problem that requires a great emphasis and some factors like smoking habit are modifiable.

## Figures and Tables

**Table 1:** Sociodemographic characteristics of respondents in Ebinat town, Northwest Ethiopia, 2015.

Variable	Category	Frequency (*n*)	Percentage (%)
Age	18–24	234	30
	25–34	298	38.3
	35–44	131	16.8
	45–54	54	6.9
	≥55	62	8

Sex	Male	416	53.4
	Female	363	46.6

Ethnicity	Amhara	775	99.5
	Tigre	4	0.5

Educational status	Unable to read and write	204	26.2
	Primary	151	19.4
	Secondary and above	424	54.4

Religion	Orthodox	682	87.5
	Muslim	95	12.2
	Protestant	2	0.3

Marital status	Widowed & divorced	106	13.6
	Single	300	38.5
	Married	373	47.6

Living circumstance	With family	561	72
	Alone	204	26.2
	Other	14	1.8

Wealth index	Lower income	141	18.1
	Medium income	382	49
	High income	256	32.9

**Table 2:** Bivariate and multivariate analysis of depression among adults of Ebinat town, Northwest Ethiopia, 2015.

Variable	Category	Depression		COR 95% CI	AOR 95% CI
		Yes	No		
Sex	Male	45	371	1	1
	Female	91	272	2.75 (1.86–4.07)	**2.45 (1.40–4.29)** ^*∗∗*^

Age	18–24	25	209	1	1
	25–34	46	252	1.52 (0.90–2.56)	1.82 (0.95–3.45)
	35–44	30	101	2.48 (1.38–4.44)	3.44 (1.45–8.18)
	45–54	10	44	1.90 (0.85–4.23)	2.04 (0.69–6.07)
	55 & above	25	37	5.64 (2.97–10.87)	**5.21 (1.89–14.37)** ^*∗∗*^

Marital status	Widowed & divorced	47	59	7.45 (4.45–12.47)	**2.60 (1.28–5.27)** ^*∗∗*^
	Single	53	247	2.00 (1.27–3.16)	**2.10 (1.08–4.06)** ^*∗*^
	Married	36	337	1	1

Educational status	Unable to read & write	53	151	2.45 (1.60–3.75)	0.67 (0.31–1.41)
	Primary	30	121	1.73 (1.06–2.84)	1.04 (0.54–1.99)
	Secondary & above	53	371	1	1

Occupation	Employed	54	409	1	1
	Nonemployed	82	234	2.65 (1.817–3.87)	0.73 (0.41–1.28)

Social support	Poor	99	179	10.23 (4.82–21.74)	**7.40 (3.16–17.32)** ^*∗∗∗*^
	Moderate	29	316	1.70 (0.75–3.80)	1.83 (0.76–4.40)
	Strong	8	148	1	1

Current tobacco use (smoking)	Yes	6	9	3.25 (1.13–9.29)	**5.67 (1.47–21.47)** ^*∗*^
	No	130	634	1	1

Comorbid medical illness	Yes	35	23	9.34 (5.30–16.46)	**7.22 (3.51–14.88)** ^*∗∗∗*^
	No	101	620	1	1

Living circumstance	Alone	64	140	3.105 (2.11–4.56)	**2.58 (1.50–4.44)** ^*∗∗*^
	Other	0	14		
	Family	72	489	1	1

Wealth index	Lower	47	94	3.76 (2.245–6.31)	2.19 (1.14–4.23)^*∗*^
	Medium	59	323	1.37 (0.85–2.20)	0.83 (0.46–1.50)
	Higher	30	226	1	1

*Note*. ^*∗*^ *p *< 0.05, ^*∗∗*^ *p *< 0.01, and ^*∗∗∗*^ *p *< 0.001. COR = crude odds ratio at 95% confidence interval;  AOR = adjusted odds ratio at 95% confidence interval.
